# Supplementary material for: Multidimensional evaluation of large language models on the AAP in-service examination: Assessing accuracy, calibration, and citation reliability
Source: PLOS Digit Health. 2026 May 29;5(5):e0001072. doi: 10.1371/journal.pdig.0001072 (PMC13220994; doi:10.1371/journal.pdig.0001072)
Supplement: S1 Text — Full documentation of all standardized prompts used for querying GPT-4.0, GPT-5.0, and Claude Sonnet 4.0 during the evaluation. (DOCX) [file pdig.0001072.s001.docx]

**COMPLETE PROMPT DOCUMENTATION**

1. **Experimental Conditions Overview**

- **GPT-4.0**: Full Test (FT) + Individual Question (IQ) — Answer only
- **GPT-5.0**: Full Test (FT) + Individual Question (IQ) — Answer + Confidence + Citations
- **Claude Sonnet 4.0**: Individual Question (IQ) only — Answer + Confidence + Citations

1. **Access Method**

- All models accessed through consumer web-based chat interfaces
- GPT-4.0 and GPT-5.0: ChatGPT Plus subscription (chat.openai.com)
- Claude Sonnet 3.5: Claude Pro subscription (claude.ai)

1. **Model Parameters**

- All models used default platform settings
- Temperature: Platform default
- Top-p, top-k, frequency penalty: Not modified
- No custom system prompts or instructions beyond the user prompts described below

1. **Full Test (FT) Implementation Details**

- All 331 questions presented in single conversation thread
- Questions formatted as numbered list with answer choices clearly labeled
- Entire examination fit within standard context window
- Model generated sequential responses for all questions in one output

1. **Individual Question (IQ) Implementation Details**

- Each of 331 questions were presented one at a time in sequential order within the same conversation thread.
- After the model provided a response to each question, the next question was presented in the subsequent user message within the ongoing conversation
- No information carried over between questions (eliminated in-context learning effects)
- Each conversation contained only: (1) task instruction + (2) single question with choices

**1. GPT-4.0 PROMPTS (Answer Only)**

1. **GPT-4.0 Full Test (FT) Format**

**User Prompt:**

You will be presented with 331 multiple-choice questions from a periodontal specialty examination. For each question, select the single most accurate answer choice using all relevant knowledge and sources available to you.

Provide your responses in the following format:

Q1: [Letter A-E]

Q2: [Letter A-E]

Q3: [Letter A-E]

...

Q331: [Letter A-E]

[COMPLETE EXAMINATION WITH ALL 331 QUESTIONS FOLLOWS]

Question 1:

[Question stem]

A. [Option A]

B. [Option B]

C. [Option C]

D. [Option D]

E. [Option E]

[Continue through Q331...]

1. **GPT-4.0 Individual Question (IQ) Format**

**User Prompt** (repeated 331 times in separate conversations):

Select the single most accurate answer choice for the following multiple-choice question using all relevant knowledge and sources available to you.

Question:

[Question stem]

A. [Option A]

B. [Option B]

C. [Option C]

D. [Option D]

E. [Option E]

**2. GPT-5.0 PROMPTS (Answer + Confidence + Citations)**

1. **GPT-5.0 Full Test (FT) Format**

You will be presented with 331 multiple-choice questions from a periodontal specialty examination. For each question:

1. Select the single most accurate answer choice using all relevant knowledge and sources available to you

2. Indicate your confidence in the selected answer as a percentage from 0-100%, where 0% means completely uncertain and 100% means completely certain

3. Provide one or more verifiable citations that support your response choice, including Digital Object Identifiers (DOIs), PubMed identifiers (PMIDs), or persistent URLs when available

Provide your responses in the following format:

Q1: [Letter A-E] | Confidence: [0-100] % | Citation(s): [Full reference with DOI/PMID/URL]

Q2: [Letter A-E] | Confidence: [0-100] % | Citation(s): [Full reference with DOI/PMID/URL]

Q3: [Letter A-E] | Confidence: [0-100] % | Citation(s): [Full reference with DOI/PMID/URL]

...

Q331: [Letter A-E] | Confidence: [0-100] % | Citation(s): [Full reference with DOI/PMID/URL]

[COMPLETE EXAMINATION WITH ALL 331 QUESTIONS FOLLOWS]

Question 1:

[Question stem]

A. [Option A]

B. [Option B]

C. [Option C]

D. [Option D]

E. [Option E]

[Continue through Q331...]

1. **GPT-5.0 Individual Question (IQ) Format**

Select the single most accurate answer choice for the following multiple-choice question using all relevant knowledge and sources available to you.

Question:

[Question stem]

A. [Option A]

B. [Option B]

C. [Option C]

D. [Option D]

E. [Option E]

After providing your answer, also:

1. Indicate your confidence in this answer as a percentage from 0-100%, where 0% means completely uncertain and 100% means completely certain

2. Provide one or more verifiable citations that support your selected answer. Include complete reference information with DOI, PMID, or persistent URL when available

Format your response as:

Answer: [Letter A-E]

Confidence: [0-100] %

Citation(s): [Full reference with DOI/PMID/URL]

**3. CLAUDE SONNET 4.0 PROMPTS (Answer + Confidence + Citations)**

1. **Claude Sonnet 4.0 Individual Question (IQ) Format ONLY**

**System/Context:** None (fresh conversation for each question)

**User Prompt** (repeated 331 times in separate conversations):

Select the single most accurate answer choice for the following multiple-choice question using all relevant knowledge and sources available to you.

Question:

[Question stem]

A. [Option A]

B. [Option B]

C. [Option C]

D. [Option D]

E. [Option E]

After providing your answer, also:

1. Indicate your confidence in this answer as a percentage from 0-100%, where 0% means completely uncertain and 100% means completely certain

2. Provide one or more verifiable citations that support your selected answer. Include complete reference information with DOI, PMID, or persistent URL when available

Format your response as:

Answer: [Letter A-E]

Confidence: [0-100] %

Citation(s): [Full reference with DOI/PMID/URL]
