# Supplementary material for: Multidimensional evaluation of large language models on the AAP in-service examination: Assessing accuracy, calibration, and citation reliability
Source: PLOS Digit Health. 2026 May 29;5(5):e0001072. doi: 10.1371/journal.pdig.0001072 (PMC13220994; doi:10.1371/journal.pdig.0001072)
Supplement: S2 Text — Definitions and descriptions of all variables included in the study dataset. (DOCX) [file pdig.0001072.s002.docx]

**Supplementary File: Data Dictionary**

**Dataset Description**

This dataset contains all AI model responses, confidence scores, citations, and validation results for 331 questions from the 2024 American Academy of Periodontology In-Service Examination across five experimental conditions.

**Column Definitions**

**Question Identifiers**

- **Question_ID**: Unique identifier for each examination question (Q001–Q331)
- **Topic_Section**: Content area as labeled in the AAP examination
  - Embryology/Anatomy/Biostatistics
  - Biochemistry/Physiology
  - Microbiology/Immunology
  - Periodontal Etiology/Pathogenesis
  - Pharmacology/Therapeutics
  - Diagnosis
  - Treatment Planning/Prognosis
  - Therapy
  - Oral Pathology/Medicine

**Answer Data**

- **AAP_Answer**: Correct answer as provided by the American Academy of Periodontology (A, B, C, D, or E)

**Model Response Variables**

**Model Naming Convention:**

- **4.0 FT** = ChatGPT-4.0, Full Test format (all questions presented simultaneously)
- **4.0 IQ** = ChatGPT-4.0, Individual Question format (one question per conversation)
- **5.0 FT** = ChatGPT-5.0, Full Test format
- **5.0 IQ** = ChatGPT-5.0, Individual Question format
- **Claude** = Claude Sonnet 3.5, Individual Question format

**For Each Model:**

- **[Model]_Response**: AI-selected answer (A, B, C, D, or E)
- **[Model]_Accuracy**: Response correctness based on matching AAP answer
  - 1 = Correct (model answer matches AAP answer)
  - 0 = Incorrect (model answer does not match AAP answer)

**Confidence Scores (GPT-5.0 and Claude only)**

- **[Model]_Confidence**: Self-reported confidence level elicited via prompt
  - Scale: 0–100
  - 0 = Completely uncertain
  - 100 = Completely certain
  - *Note: GPT-4.0 models did not provide confidence scores*

**Citation Variables (GPT-5.0 and Claude only)**

**Citation Type:**

- **[Model]_Citation_Type**: Format of source cited
  - 1 = Journal article
  - 2 = Book/textbook
  - 3 = Webpage/online resource
  - Blank = No citation provided

**Citation Validity:**

- **[Model]_Citation_Validity**: Whether citation exists and is verifiable
  - 1 = Valid (citation exists and is verifiable)
  - 0 = Invalid (citation is fabricated, unverifiable, or critically wrong)

**Citation Taxonomy:**

- **[Model]_Citation_Taxonomy**: Detailed validity classification based on human expert verification
  - **1 = Critically Wrong/Unverifiable/Fabricated**: Citations that cannot be located in any database, include fabricated authors, non-existent journals, fake DOIs, or invented chapter/book titles
    - *Example*: "Martinez-Rodriguez D, Williams GH. Biomaterial innovations in periodontal surgery. Journal of Periodontal Biomaterials. 2019;42(3):156-174. DOI: 10.1902/JPER.2019.0456" — Journal does not exist; authors not found; DOI non-functional
  - **2 = Partially Correct with Minor Errors**: Real citations with minor bibliographic inaccuracies such as incorrect page numbers (off by 1-3 pages), minor spelling variations in author names, or wrong volume/issue numbers
    - *Example*: "Cortellini P, Tonetti MS. Clinical concepts for regenerative therapy in intrabony defects. Periodontol 2000. 2015;68:282-307." — Citation exists; correct authors/year/journal; actual page range is 282-299 (off by 8 pages)
  - **3 = Verified and Correct**: Citations that exist, are accessible, contain accurate bibliographic information (authors, year, journal, volume, pages), and the content directly supports the model's answer
    - *Example*: "Needleman I, Worthington HV, Giedrys-Leeper E, Tucker R. Guided tissue regeneration for periodontal infra-bony defects. Cochrane Database Syst Rev. 2006;(2):CD001724. DOI: 10.1002/14651858.CD001724.pub2" — Fully verified; all details accurate; content supports response.
