# Supplementary material for: Multidimensional evaluation of large language models on the AAP in-service examination: Assessing accuracy, calibration, and citation reliability
Source: PLOS Digit Health. 2026 May 29;5(5):e0001072. doi: 10.1371/journal.pdig.0001072 (PMC13220994; doi:10.1371/journal.pdig.0001072)
Supplement: S3 Text — Detailed protocol used for expert review of model-generated citations and hallucination classification. (DOCX) [file pdig.0001072.s003.docx]

**Human-in-the-Loop Citation Validation protocol**

1. **Citation Verification Methodology**

All citations generated by GPT-5.0 and Claude Sonnet 3.5 underwent systematic human verification to assess validity and hallucinated or fabricated references.

​a) **Step 1: Database Retrieval**
Citations were searched in multiple academic databases:

- PubMed (pubmed.ncbi.nlm.nih.gov)
- Google Scholar (scholar.google.com)
- Web of Science
- Publisher-specific databases (Wiley, Elsevier, Springer, etc.)

Search strategies included exact title matching, author name combinations, and partial citation elements when complete citations could not be immediately located.

**b) Step 2: Identifier Verification**
When citations included Digital Object Identifiers (DOIs) or PubMed Identifiers (PMIDs), these were verified against official registries:

- DOI resolution tested via doi.org
- PMID verification via PubMed database

Non-functional identifiers (DOIs that did not resolve or PMIDs not found in PubMed) were flagged as fabricated.

**c) Step 3: Bibliographic Accuracy Assessment**
For successfully located citations, the following elements were cross-checked against authoritative source records:

- Author names (spelling, order, completeness)
- Publication year
- Journal or book title
- Volume and issue numbers
- Page ranges

**d)** **Step 4: Contextual Relevance Assessment**

After verifying bibliographic accuracy (Steps 1-3), each citation underwent contextual relevance evaluation. One investigator with domain expertise in periodontology assessed whether each bibliographically valid citation was substantively relevant to the specific question it was used to support.
Citations that were non relevant to the question were considered invalid.

1. **Three-Tier Classification Taxonomy**
   1. **Category 1: Completely Valid**

Citation exists in academic databases, all bibliographic details are accurate, and DOI/PMID are accurate and functional. Contextually relevant to the question topic.

Example:

- Needleman IG, Worthington HV, Giedrys-Leeper E, Tucker RJ. Guided tissue regeneration for periodontal infra-bony defects. Cochrane Database Syst Rev. 2006 Apr 19;(2):CD001724. doi: 10.1002/14651858.CD001724.pub2. Update in: Cochrane Database Syst Rev. 2019 May 29;5:CD001724.

doi: 10.1002/14651858.CD001724.pub3. PMID: 16625546.

- Verification Result: ✓ Valid
- - DOI resolves correctly to Cochrane Library
- - All authors verified in correct order
- - All bibliographic details accurate

**b) Category 2: Partially Correct**

Citation refers to a real, locatable source, but contains minor bibliographic inaccuracies such as page number discrepancies (±1-10 pages), author name spelling variations, or volume/issue errors. Contextually relevant to the question topic.

Example:

Cortellini P, Tonetti MS. Clinical concepts for regenerative therapy in intrabony defects. Periodontol 2000. 2015;68:299-307.

Verification Result: Partially Correct

- Article exists and was located

- Authors, journal, year, volume all correct

- ACTUAL page range: 68:282-307 (off by 17 pages)

**c) Category 3: Invalid**

Citation cannot be located in any academic database, contains fabricated elements (non-existent journals, fictitious authors, invalid DOIs), or refers to unverifiable sources or contextually irrelevant.

Example:

Martinez-Rodriguez D, Williams GH. Advanced biomaterials in periodontal regeneration. Journal of Periodontal Biomaterials. 2019;42(3):156-174. DOI: 10.1902/JPER.2019.0456

Verification Result: Critically Wrong

- "Journal of Periodontal Biomaterials" does not exist in any academic database

- Authors not found with this publication

- DOI does not resolve

- Completely fabricated

- Contextually irrelevant
